# Interrupting seasonal transmission of *Schistosoma haematobium* and control of soil-transmitted helminthiasis in northern and central Côte d’Ivoire: a SCORE study protocol

**DOI:** 10.1186/s12889-018-5044-2

**Published:** 2018-01-29

**Authors:** Yves-Nathan T. Tian-Bi, Mamadou Ouattara, Stefanie Knopp, Jean T. Coulibaly, Eveline Hürlimann, Bonnie Webster, Fiona Allan, David Rollinson, Aboulaye Meïté, Nana R. Diakité, Cyrille K. Konan, Eliézer K. N’Goran, Jürg Utzinger

**Affiliations:** 1 Unité de Formation et de Recherche Biosciences, Université Félix Houphouët-Boigny, 22 BP 770, Abidjan, 22 Côte d’Ivoire; 2 Centre Suisse de Recherches Scientifiques en Côte d’Ivoire, 01 BP 1303, Abidjan, 01 Côte d’Ivoire; 3 Swiss Tropical and Public Health Institute, P.O. Box, CH–4002 Basel, Switzerland; 4 University of Basel, P.O. Box, CH–4003 Basel, Switzerland; 5 Wolfson Wellcome Biomedical Laboratories, Department of Life Sciences, Natural History Museum, Cromwell Road, London, SW7 5BD United Kingdom; 6 Programme National de Lutte contre les Maladies Tropicales Négligées à Chimiothérapie Préventive, Ministère de la Santé et de l’Hygiène Publique, 06 BP 6394, Abidjan, 06 Côte d’Ivoire

**Keywords:** *Bulinus* spp., Chemical snail control, Côte d’Ivoire, Mass drug administration, Niclosamide, Praziquantel, *Schistosoma haematobium*, Seasonal transmission, Soil-transmitted helminthiasis

## Abstract

**Background:**

To achieve a world free of schistosomiasis, the objective is to scale up control and elimination efforts in all endemic countries. Where interruption of transmission is considered feasible, countries are encouraged to implement a comprehensive intervention package, including preventive chemotherapy, information, education and communication (IEC), water, sanitation and hygiene (WASH), and snail control. In northern and central Côte d’Ivoire, transmission of *Schistosoma haematobium* is seasonal and elimination might be achieved. In a cluster-randomised trial, we will assess different treatment schemes to interrupt *S. haematobium* transmission and control soil-transmitted helminthiasis over a 3-year period. We will compare the impact of (i) arm A: annual mass drug administration (MDA) with praziquantel and albendazole before the peak schistosomiasis transmission season; (ii) arm B: annual MDA after the peak schistosomiasis transmission season; (iii) arm C: two yearly treatments before and after peak schistosomiasis transmission; and (iv) arm D: annual MDA before peak schistosomiasis transmission, coupled with chemical snail control using niclosamide.

**Methods/design:**

The prevalence and intensity of *S. haematobium* and soil-transmitted helminth infections will be assessed using urine filtration and Kato-Katz thick smears, respectively, in six administrative regions in northern and central parts of Côte d’Ivoire. Once a year, urine and stool samples will be collected and examined from 50 children aged 5–8 years, 100 children aged 9–12 years and 50 adults aged 20–55 years in each of 60 selected villages. Changes in *S. haematobium* and soil-transmitted helminth prevalence and intensity will be assessed between years and stratified by intervention arm. In the 15 villages randomly assigned to intervention arm D, intermediate host snails will be collected three times per year, before niclosamide is applied to the selected freshwater bodies. The snail abundance and infection rates over time will allow drawing inference on the force of transmission.

**Discussion:**

This cluster-randomised intervention trial will elucidate whether in an area with seasonal transmission, the four different treatment schemes can interrupt *S. haematobium* transmission and control soil-transmitted helminthiasis. Lessons learned will help to guide schistosomiasis control and elimination programmes elsewhere in Africa.

**Trial registration:**

**ISRCTN** ISRCTN10926858. Registered 21 December 2016. Retrospectively registered.

## Background

### Burden and transmission of schistosomiasis and soil-transmitted helminthiasis

More than a billion people are affected by schistosomiasis and soil-transmitted helminthiasis worldwide [1]. An estimated 300 million people with heavy parasitic worm infections have measurable morbidity, among them, more than 50% are school-aged children [2, 3]. In 2013, the global burden due to schistosomiasis and soil-transmitted helminthiasis was estimated at 7.09 million disability-adjusted life years (DALYs) [4].

Schistosomiasis is a water-associated disease caused by chronic infection with parasitic trematodes of the genus *Schistosoma*. The life cycle of these blood-dwelling flukes involves a definitive human host, in which adult schistosome worms live in the perivesical or mesenteric venous plexus of their human host. These worms live as couples and sexually reproduce and shed eggs. Specific freshwater snails are infested by miracidia hatched from the eggs of adult worms and the snails act as intermediate host, in which asexual multiplication of the parasite takes place and from which infectious larval stages (cercaria), are shed into water. Infection occurs when humans make contact with freshwater bodies harbouring these cercaria [5]. The disease schistosomiasis is caused primarily by inflammatory reactions due to the deposition of schistosome eggs trapped in tissues surrounding the bladder or intestines. Urogenital schistosomiasis, in which the bladder, genital tract and urethras are affected, is caused by infection with the species *S. haematobium*, which occurs mainly in Africa.

Soil-transmitted helminthiasis is caused by a group of intestinal nematode worms, the most important of which are the anthropophilic hookworm (*Ancylostoma duodenale* and *Necator americanus*), roundworm (*Ascaris lumbricoides*) and whipworm (*Trichuris trichiura*). The life cycles of hookworm, *A. lumbricoides* and *T. trichiura* follow a general pattern. The adult parasite stages inhabit the gastrointestinal tract, reproduce sexually and produce eggs, which are passed in human faeces and are deposited in the external environment. Larval stages hatch from the eggs either in the soil (hookworm) or in the intestines after ingestion of food or water contaminated with eggs (*A. lumbricoides* and *T. trichiura*). To reach the adult stage, the larvae can migrate into the human body (after passing through the skin or the digestive mucosa) and return to the intestine (*A. lumbricoides* or hookworm) or remain there (*T. trichiura*) [6]. Clinical complications are mainly related to the chronic and insidious effects on the hosts’ health and nutritional status [7]. Hookworm infections have long been recognised as an important cause of intestinal blood loss leading to iron deficiency and protein malnutrition. Indeed, iron deficiency anaemia accompanying moderate and heavy hookworm burdens is sometimes referred to as “hookworm disease” [8]. Chronic soil-transmitted helminth infections impact the physical and mental development of children [9].

### *S. haematobium* and soil-transmitted helminth infections in Côte d’Ivoire

**Fig. 1:**
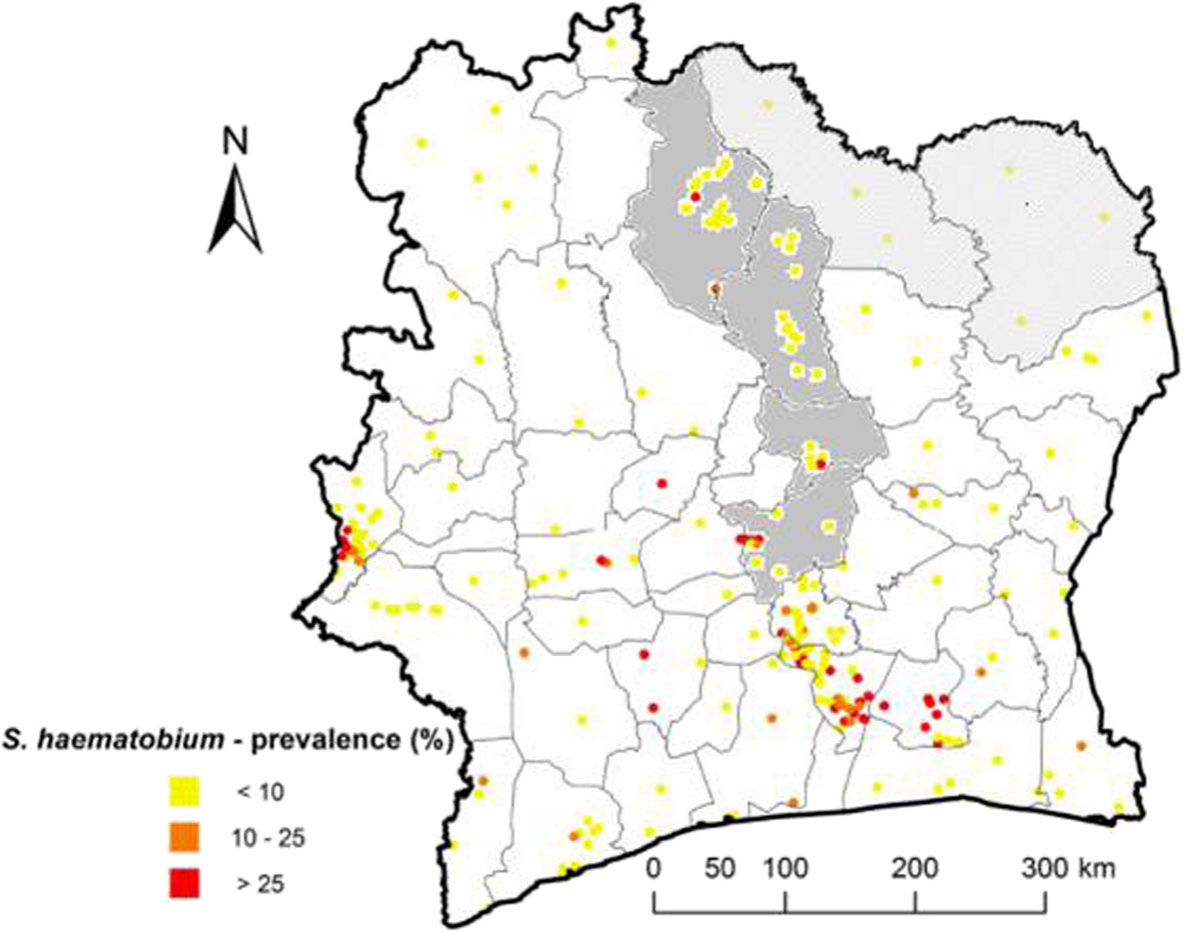
Map of Côte d’Ivoire, showing geo-referenced *S. haematobium* prevalence survey data. Source: Global Neglected Tropical Diseases database, www.gntd.org [16]. The districts included into the study are highlighted in grey in the central part and with grey hachures in the northern part of Côte d’Ivoire

A number of important points regarding the epidemiology and ecology of schistosomiasis and soil-transmitted helminthiasis are worth highlighting for the northern and central parts of Côte d’Ivoire. First, *S. haematobium* is the predominant *Schistosoma* species [10–12]. Historical data and recent empiric studies revealed a low to moderate prevalence of urogenital schistosomiasis in northern and central Côte d’Ivoire, with a *S. haematobium* prevalence rarely found in excess of 10% among school-aged children [13–17] (Fig. 1). Preliminary data obtained in early 2014 during a rapid appraisal mapping done by our group in two regions (Bounkani and Tchologo) in northern Côte d’Ivoire revealed a prevalence of 3.3–26.7% for *S. haematobium* and 3.3% for *S. mansoni* [15].

**Fig. 2:**
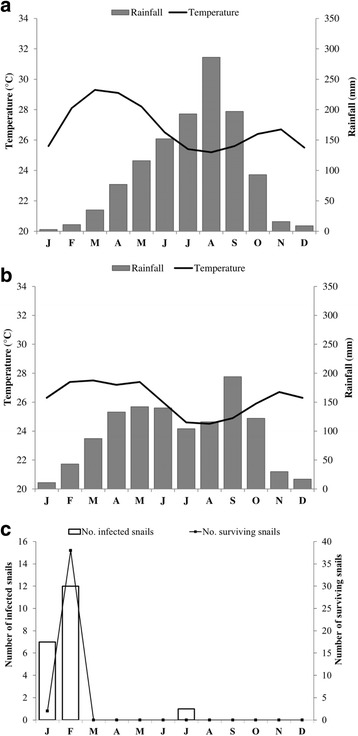
Climate diagrams (rainfall and temperature) in (**a**) northern and (**b**) central Côte d’Ivoire. Adapted from climate-data.org [17] (**c**) *S. haematobium* transmission dynamics in Tiengarakaha (Ferkessedougou), in the northern part of Côte d’Ivoire. Comparison of numbers of *B. truncatus* snails infected (left y-axis) and surviving (right y-axis) collected from the lake of Tiengarakaha 30 days after the maintenance of snails in the laboratory. Adapted from N’Goran [11]

Two species of freshwater snails (*Bulinus truncatus* and *B. globosus*) transmit urogenital schistosomiasis in Côte d’Ivoire [10–12]. In the northern part of Côte d’Ivoire, the major intermediate host snail for *S. haematobium* is *B. truncatus*, whereas both snail species are involved in transmission in the central part of the country. Due to two distinct seasons, with rainfall occurring between March and October and the dry period lasting from November to March, there is a strong seasonality in the presence of snails and schistosomiasis transmission [11] (Fig. 2a and b). The abundance of *S. haematobium*-infected intermediate host snails is strongly driven by this seasonality. Indeed, infected snails have exclusively been identified during the dry season (January and February) in man-made stagnant freshwater bodies [11, 18] (Fig. 2c). Soil-transmitted helminths are in general more widely distributed in Côte d’Ivoire and transmitted throughout the year. In a recent national survey among school-aged children, the prevalence of hookworm, *A. lumbricoides* and *T. trichiura* was 17.2%, 1.9% and 1.3%, respectively [15].

### Control of schistosomiasis and soil-transmitted helminthiasis in Côte d’Ivoire

The life cycle of *S. haematobium* can be interrupted by: (i) killing the worms in humans, using anthelminthic drugs; (ii) killing the intermediate host snails using chemicals (i.e. molluscicides), biological control agents (e.g. competitor snails, fish and prawns) or environmental management (e.g. draining of swamps); (iii) sensitizing people not to urinate into open freshwater bodies so that intermediate host snails cannot become infected; and (iv) keeping people out of infested water bodies to avoid infection of humans with cercaria [18–21].

The global strategy to control schistosomiasis is based on morbidity control through preventive chemotherapy that is the periodic administration of praziquantel to at-risk populations (e.g. school-aged children) without prior individual diagnosis [22]. In Côte d’Ivoire, there is a history of focal control interventions that were implemented in the southern, central and western regions, starting in the 1990s [23]. Thereafter, Côte d’Ivoire has gone through a decade of socio-political crisis that has degraded the health system, as well as control interventions targeting schistosomiasis and other neglected tropical diseases [24]. In late 2010, the managers of six neglected tropical diseases control programmes developed a 5-year integrated operational plan, readily assisted by experts from the World Health Organization (WHO). These control programmes include (i) the national control programme for schistosomiasis, soil-transmitted helminthiasis and lymphatic filariasis and (ii) the national programme for eye health and control of onchocerciasis. The 5-year operational plan is currently awaiting full endorsement by national and international experts. Meanwhile, mass drug administration (MDA) for schistosomiasis has commenced in 2013 with an initial emphasis on the Cavaly, Guemon and Tonkpi regions in western Côte d’Ivoire [25–27] and on the Agnéby region in south-eastern Côte d’Ivoire [28]. Moreover, to better coordinate the control of these diseases in Côte d’Ivoire, a national programme for the control of neglected tropical diseases with preventive chemotherapy (Programme National de Lutte contre les Maladies Tropicales Négligées à Chimiothérapie Préventive; PNLMTN-CP) has been initiated in December 2016. The programme is supported by the Schistosomiasis Consortium for Operational Research and Evaluation (SCORE) and the Schistosomiasis Control Initiative (SCI).

### Operational research for schistosomiasis control in Côte d’Ivoire

According to WHO, helminthiasis control programmes should combine the administration of praziquantel against schistosomiasis and albendazole/mebendazole against soil-transmitted helminthiasis, as these infections often overlap geographically [29]. Wherever elimination of schistosomiasis seems feasible, countries are encouraged to change their control to an elimination strategy and to provide praziquantel in intervals shorter than 12 months and to complement with snail control and behaviour change interventions, when applicable in order to interrupt disease transmission [30].

In line with WHO considerations to shift from morbidity control to elimination of schistosomiasis as a public health problem, the SCORE project described here will focus on the interruption of *S. haematobium* transmission. The study is carried out in the northern and central parts of Côte d’Ivoire, where schistosomiasis transmission is seasonal. Specifically, we will assess the impact of different interventions in four study arms: (i) arm A: MDA before the peak transmission season of schistosomiasis; (ii) arm B: annual MDA after the peak schistosomiasis transmission season; (iii) arm C: two yearly MDA treatments before and after the peak schistosomiasis transmission; and (iv) arm D: annual MDA before the peak schistosomiasis transmission, coupled with tri-annual chemical snail control.

This is a 3-year randomised intervention study, preceded by one year of initial eligibility and baseline surveys conducted in 2015, which allowed the selection of 60 study villages where interventions are implemented. The impact of the interventions in each study arm will be assessed in annual follow-up surveys carried out in late 2016, late 2017 and late 2018.

The collaborative project involves teams from the Université Félix Houphouët-Boigny (UFHB) and the Centre Suisse de Recherches Scientifiques en Côte d’Ivoire (CSRS; both based in Abidjan, Côte d’Ivoire), the Swiss Tropical and Public Health Institute (Swiss TPH), an associated institute of the University of Basel; (Basel, Switzerland) and the Natural History Museum (NHM; London, United Kingdom). The project will be implemented in close collaboration with the PNLMTN-CP and linked to ongoing large-scale schistosomiasis control efforts in the country, supported by SCI.

## Methods/design

### Goal, aims and objectives

The goal of this SCORE study is to provide an evidence-base for programme decisions about interrupting the transmission of *S. haematobium* in a setting where transmission is seasonal. In a cluster-randomised intervention trial, we will determine whether different treatment schemes can interrupt *S. haematobium* transmission and whether soil-transmitted helminthiasis can be controlled. The study will run for 3 years and includes one year of eligibility and a detailed baseline survey. Each intervention arm will include activities in 15 villages, hence a total of 60 communities.

The aims of the projects are (i) to interrupt seasonal transmission of *S. haematobium* in pre-selected areas in northern and central Côte d’Ivoire within 3 years of interventions; and (ii) to control soil-transmitted helminthiasis in the same communities. There are four specific objectives, activities and milestones, as follows:

- to assess annually the prevalence and intensity of *S. haematobium* and soil-transmitted helminth infections in communities (children and adults), according to age and sex, and within and between each study arm from year 1 to year 3;
- to determine the coverage and compliance with annual or biannual MDA with praziquantel for *S. haematobium* and albendazole for soil-transmitted helminth infections;
- to assess the impact of chemical snail control with niclosamide on the presence of *Bulinus* spp. snails and on non-target fauna in freshwater bodies; and
- to identify the different schistosome species of cercaria shed by infected *Bulinus* spp. snails using molecular methods.

### Study setting

The study will be carried out in six administrative regions of Côte d’Ivoire, four of which are located in the north (Tchologo, Bounkani, Poro and Hambol) and two in the centre of the country (Gbêkê and Bélier). These regions have virtually the same climate and soil characteristics. The Köppen-Geiger climate classification [31] qualifies the climate of these regions as an “Aw type”; hence, a tropical and dry or savannah climate. In northern Côte d’Ivoire, the temperature ranges from 25 °C to 29 °C (mean: 26.8 °C) and the monthly precipitation varies between 3 mm and 300 mm (annual mean: 1300 mm). In the central part, the values are 24 °C to 27 °C (mean: 26.2 °C) and 11 mm to 200 mm (annual mean: 1100 mm), respectively (https://fr.climate-data.org). The dry season lasts from November to March, though a quite high rainfall is recorded in March in the central part of the country (see Fig. 2a and b). Of note, in December and January, the Harmattan – a strong dry wind from the Sahara – greatly lowers the temperature, particularly at night. The soils are mostly tropical ferruginous soils.

### Population and primary economic activities

The Tchologo region is composed of three departments and the Bounkani region is subdivided into four departments. The Poro region consists of four departments, while the Hambol region has three departments. The Gbêkê and Bélier regions have four departments each. In those regions three quarters of the population are aged below 40 years [32].

People are mainly engaged in subsistence farming (e.g. maize, millet, sorghum and yam). The two main cash crops are cotton and cashew. Moreover, there are some irrigated rice farming and vegetable farming and fishing activities in small multi-purpose dams, which were initially constructed for livestock breeding. Indeed, Tchologo and Bounkani regions are suitable areas for livestock breeding. During the dry season, there is a significant reduction of water sources and surfaces, but with some remaining water points in the dams. This results in increased contact of populations with these sites in the dry periods, and hence, an increased risk of transmission of water-related parasitic diseases such as schistosomiasis.

### Justification of the number of clusters and participants

The selection of four intervention arms for this study was based on equilibrium between what is scientifically and technically recommended and what is practically feasible during the eligibility and follow-up surveys. Sixty study villages have been selected with 15 villages in each of the four intervention arms. To assess the (baseline) epidemiological situation of helminth infections in an area, the WHO recommends sampling of at least 50 children in one school [3]. In the randomised intervention trial, we aimed to include a total of 48,000 participants (i.e. 50 + 100 + 50 = 200 participants * 60 villages * 4 surveys).

### Eligibility of study communities and randomisation

Villages were eligible to be included into the study if they fulfilled the following criteria:

- the village is located in an area with a well-defined *S. haematobium* transmission season;
- there are at least 100 children aged 9–12 years enrolled at school (determined by readily available school lists); and
- the prevalence of *S. haematobium* is at least 4%, as determined by a single mid-day urine filtration of 50 children aged 13–14 years in a prior eligibility survey.

**Fig. 3:**
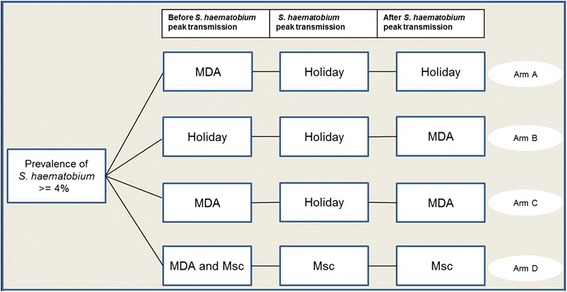
Cluster randomised intervention trial with four intervention arms. The study will be implemented over a 3-year period, and hence, the interventions specified here will be repeated every year. MDA, mass drug administration; Holiday, no intervention; Msc, mollusciciding

Once 60 eligible villages were identified, they were randomly assigned to one of four intervention arms (Fig. 3):

- arm A: annual MDA with praziquantel and albendazole before the peak transmission season of schistosomiasis (November/December);
- arm B: annual MDA with praziquantel and albendazole after the peak transmission season of schistosomiasis (March/April);
- arm C: two yearly MDAs before and after the peak transmission of schistosomiasis (November/December and March/April); and
- arm D: annual MDA with praziquantel and albendazole before the peak transmission season of schistosomiasis (November/December), coupled with chemical snail control using niclosamide (three applications per year: before, during and shortly after the peak transmission).

### Selection of study villages

To identify 60 eligible study communities, we pursued a two-stage approach, making use of the existing education system. A pre-screening for potential study villages was conducted by applying a rapid-assessment questionnaire for the presence of blood in urine that served as a proxy for *S. haematobium* infection. Of note, this questionnaire has previously been validated in Côte d’Ivoire [33]. Teachers from the study regions were invited to interview children attending grades 3–5. The schools with a *S. haematobium* prevalence above 4% of reported blood in urine during the 2 weeks preceding the questionnaire were selected for further investigation. In eligible schools, 50 children aged 13–14 years were randomly selected and invited to provide a single mid-day urine sample. The urine samples were processed by a filtration method for detection of *S. haematobium* eggs [34]. In addition, urine samples were subjected to reagent strip testing for microhaematuria (a proxy for *S. haematobium* infection) [35] and a point-of-care circulating cathodic antigen (POC-CCA) cassette test for the diagnosis of *S. mansoni* [36]. Once 60 villages with a prevalence of *S. haematobium* ≥ 4%, based on urine filtration, were identified, they were included in the study and randomised to one of four study arms.

### Parasitological surveys

During the baseline survey prior to any intervention and at annual cross-sectional surveys conducted over a 3-year period, single urine and stool samples will be collected from 50 children aged 5–8 years, 100 children aged 9–12 years and 50 adults aged 20–55 years in each of the 60 study villages. A mid-day urine specimen (at least 20 ml) and an early morning stool sample will be collected from each participant. Urine and stool samples will be examined in the laboratory at the district health centre.

### Examination of urine and stool samples

In the laboratory, each urine sample will be tested for the presence of haematuria (visual detection for macrohaematuria and reagent strip testing for microhaematuria) [37]. *S. haematobium* eggs will be quantified by urine filtration (10 ml urine) under a microscope [37, 38]. Two microscope slides will be prepared per stool sample using the Kato-Katz technique [39]. To this end, thick smears will be prepared from each sample using standard 41.7 mg templates. The slides will be examined under a microscope as soon as prepared for the detection of hookworm eggs and a second time after clearing of at least 45 min for the detection of other soil-transmitted helminths and *S. mansoni*. Helminth eggs will be counted and recorded for each species separately [25].

### MDA and tracking compliance

Two MDA approaches will be used: school-based treatment (SBT) and community-wide treatment (CWT). Praziquantel and albendazole will be provided as a single oral dose of 40 mg/kg and 400 mg, respectively, according to the schedules defined for the different study arms. School teachers and community-drug distributors (CDDs) will deliver the drugs to the children and the community members, respectively. Medical staff will check for exclusion criteria (e.g. hypersensitivity to praziquantel or albendazole, pregnancy among female participants). In all arms of the study, praziquantel will also be provided to health centres for the management of all cases detected during routine consultations.

To estimate treatment coverage, school teachers and CDDs who will deliver the drug to the children and the community members, respectively, will fill in separate treatment records. The treatment reports from teachers and CDDs will be compiled by the local principal investigator. Tracking schoolchildren will be facilitated through the education system. With regard to school-aged children not attending school and the rest of the community, we will adopt a household approach to offer everyone a “health record”, which will include a unique identifier, the person’s name, age, sex and three open boxes corresponding to 3 years of follow-up. In the latter approach, CDDs will assist with the treatment of adults and school-aged children not attending school. CDDs will be trained to record the aforementioned information for community members. Records will remain confidential.

Of note, some of the praziquantel and albendazole stock will be kept at the health centre, in order that CDDs will be able to treat members in the community who did not receive the treatment.

### Response to treatment and reporting of adverse events

Treatment will be administered by trained teachers during SBT or CDDs during CWT. Adverse events will be monitored by the physicians and nurses of the local health facilities. Individuals who received praziquantel will be invited to report to these first-line centres in case of any adverse events after drug administration (“anything abnormal occurring in your body”). The health centre personnel will be trained on how to fill in the standard record forms (adopting a grading score) and instructed to refer to second-line hospitals any individual presenting with severe adverse events that they could not manage. Both passive and active surveillance measures will be implemented during the MDA intervention. Passive measures will be aimed at ensuring rapid medical assistance for any individuals who might experience adverse events after treatment, while active measures will also be in place to elucidate the nature, frequency and severity of adverse events.

Passive measures will be established on the treatment day and the day after; one or two health centres at the district will be kept open for 24 h and equipped with first-line emergency drugs (i.e. non-steroid anti-inflammatory drugs, anti-histaminic drugs, cortisone and intravenous fluids).

### Information, education and communication (IEC) campaign

In close collaboration with the national control programme, IEC materials (e.g. posters, pamphlets, flip charts and calendars) will be introduced during community and school mobilization sessions addressed to CDDs, teachers and community members by teams formed by the principal investigators and co-investigators.

### Training of teachers and others who will administer praziquantel and albendazole

Training programmes for two teachers and two CDDs in each community (hence, a total of 120 teachers and 120 CDDs) will be developed in order to standardise the intervention (i.e. SBT and CWT). Such training will focus on basic principles of IEC, while it will provide teachers and CDDs with a basic understanding of schistosomiasis and how to administer praziquantel and albendazole and fill-in the forms. These sessions will be delivered jointly by the health personnel and the research team.

### Approaches for reaching school-aged children not attending school

With regard to the non-enrolled school-aged children, we will adhere to an approach successfully piloted in Zanzibar, where all attending schoolchildren were encouraged to inform and bring along their non-enrolled siblings and other non-attending children from the village to the school for free treatment provided at a specific treatment day [40]. Additionally, CDDs will implement a door-to-door treatment visit in order to enhance the coverage rate of non-enrolled schoolchildren and reluctant adults. Additionally, praziquantel and albendazole will be made available at health centres.

### Process measures that will be collected

We will collect the following process measures:

- number of treatments delivered against schistosomiasis and soil-transmitted helminthiasis, by whom and whether SBT, CWT or health facility-based;
- number of teachers and CDDs trained;
- number of sessions of community education, who delivered them and how many people participated;
- number of returned reports from distributors/teachers and completeness and other quality measures;
- knowledge of schistosomiasis and soil-transmitted helminthiasis, source of knowledge, opinion about treatment campaign from children and adults included in the studies; and
- knowledge of schistosomiasis and soil-transmitted helminthiasis, source of knowledge, opinion about treatment campaign from volunteer teachers and CDDs involved in the corresponding interventions and technical questions with regards to their training.

### Coverage assessment and actions taken in case of low treatment coverage

In 2014, a general national census (GNC) has been conducted to update the demographic data of Côte d’Ivoire [32]. Preliminary results on population statistics for the study area from this census have been sought from local authorities. This database served as a reference for the calculation of treatment coverage rate. In brief, we will divide the number of treated individuals by the number of eligible school-aged children and adults living in a given village. We assume that among school-aged children, an acceptable coverage might be at least 85%. In adults the target coverage might be considerably lower, and in our view an acceptable coverage might be set at a level of 75%. If these coverage rates are not reached during the treatment campaign, we will intensify sensitisation on three levels, namely (i) community-directed; (ii) health centre-based; and (iii) school-based, and repeat the treatment campaign. In addition, meetings will be held in all study villages with local authorities. In particular, village leaders (i.e. village chiefs, leaders of youth and women’s groups) will be invited to assist with setting-specific activities in order to boost coverage rates.

### Snail control, the status of niclosamide at the study site and concerns about its use

Niclosamide is the main molluscicide registered and recommended by WHO [41], and it has been widely used in the past in different contexts and countries, notably on the African continent. Currently, it is used for the control of schistosomiasis intermediate host snails in the People’s Republic of China [42], Tunisia [43], Morocco [44], Egypt [45] and the United Republic of Tanzania (Zanzibar) [37]. To our knowledge, although niclosamide is registered in Côte d’Ivoire as a drug for human use, there is no prior experience with the use of niclosamide as molluscicide in the country. Under these circumstances, approval for exceptional use in the frame of the current research project has been sought and granted from the national committee for pesticide use (Comité Pesticides de Côte d’Ivoire, Ministère de l’Agriculture; approval no. 0163; date of approval 27 January 2015). Additionally, the field implementation will be supervised by experts from NHM in London, who currently are involved in mollusciciding activities in the frame of the Zanzibar Elimination of Schistosomiasis Transmission (ZEST) project [46]. An important issue will be to carefully document potential negative impacts of the introduction of niclosamide on non-targeted fauna in the intervention villages. Côte d’Ivoire has proven skills in vector management, i.e. larviciding of rivers against blackfly larvae (vector of onchocerciasis).

Snail control will be implemented in one of the intervention arms, hence in 15 of the 60 study villages. Snail species will be located and characterised within transmission sites. In each selected community, approximately three sites will be surveyed.

Once the randomisation of the villages has been done, a baseline malacological survey will be conducted in the surroundings of the 15 target villages, thus allowing a more detailed study on schistosomiasis transmission at sites where potential intermediate host snails occur.

After the baseline malacological survey, a first focal treatment with niclosamide (concentration of 10 g/l) will be undertaken at water contact sites found to be infested with *Bulinus* spp. (*B. truncatus* and/or *B. globosus*). In order to monitor the population dynamics of non-target aquatic organisms over time, an inventory focussing on species and abundances will be conducted before the intervention and at each following malacological survey. Members of the study team will be trained on the different application techniques of molluscicides. Based on available information on transmission and climate of the study area, mollusciciding interventions are planned three times a year: before and after the main rainy season and in the middle of the dry season. The coordination of the timely MDA and the mollusciciding activities are crucial to increase the impact on snail control and *vice versa*. In this respect, each year the mollusciciding will be carried out before or during the MDA.

Additionally, the status of snail infection by *Schistosoma* spp. will be tested with the cercarial shedding method and by molecular characterisation of schistosome species in snails. This characterisation will be carried out at NHM during year 2 of the study. The aim is to gain a deeper insight on the snail species involved in local *S. haematobium* transmission and on potential other schistosome species endemic in the region. In fact, it is conceivable that *S. bovis* and other intestinal parasites, usually found in livestock, are endemic in this zone where pastoralism is common.

### Assessment of transmission foci and intensity

Water contact sites used by community members in each village will be identified and georeferenced using hand-held global positioning system (GPS; Garmin Sery GPS MAP 62; Olathe, United States of America) devices. For the identification of water contact sites, where transmission can occur, school-aged children, who have been identified as *S. haematobium*-positive, will show our team the sites where they get in contact with water for recreational or domestic activities. Additionally, we will collect relevant information of water contact from community leaders.

Once the sites have been identified, they will be searched for intermediate host snails by four experienced fieldworkers, adhering to standard protocols (i.e. 15 min of sampling in a predefined area using a mesh scoop, constructed of kitchen sieves mounted on a 1.5 m long wooden stick). Snails will be collected manually using scoops and forceps. Although this method has some limitations (e.g. ineffectiveness at sites with difficult access and the subjectivity of the investigator), it has the advantage of being simple and represents a minimal intrusion on the environment.

This process will be implemented in each study village three times, just before niclosamide is applied (i.e. before and after the rainy season and in the middle of the dry season). Snails sampled at individual sites will be stored in containers filled with water from the originating site and transferred to a laboratory at the UFHB in Abidjan. Snails will be kept at room temperature (24–27 °C). The containers will be labelled with unique identifiers. In the laboratory, snails will be kept alive in covered water tanks at room temperature, using water from the original site. Snail species will be identified from shell morphology.

To assess the transmission level, cercarial shedding will be observed over the first 48 h after collection. All *Bulinus* spp. found will be individually inspected for infection by placing snails into 12 or 24 well plates and exposed to sunlight or artificial light for 3 to 6 h and subsequently be examined under a dissection microscope for the presence of *Schistosoma* cercaria [47]. If present, a subsample of 20–30 cercariae will be collected individually onto a Whatman FTA card (GE Healthcare Life Sciences; Amersham, United Kingdom) and sent to the NHM for molecular analysis.

Additionally, a subsample of snails (up to 50 snails per site) will be fixed in ethanol and forwarded to the Schistosomiasis Collection at the NHM in London (SCAN) for molecular diagnosis [48].

### Study outcomes

#### Analysis plan to assess outcomes of interest

In addition to parasitological data, demographic data (age, sex and occupation for adults) will be recorded from each participant. Changes in the *S. haematobium* and soil-transmitted helminth infection prevalence and intensity will be assessed for each intervention arm over time. The treatment coverage rates among children and adults will be estimated by dividing the number of participants of the respective age group treated by the total number of registered individuals of the corresponding age range in the community. Adverse events and serious adverse events will be tracked and recorded for management. Community sensitisation and mobilisation efforts, radio announcements and other IEC tools will be used to help enhancing reach-out to populations targeted for treatment in the MDA. Moreover, we will make efforts to record any major change in water, sanitation and hygiene (WASH), unusual weather (e.g. drought and floods), political instability or local changes in leadership that might affect MDA efforts, changes in the existing health system, community attitudes and behaviour related to health and health care seeking and other programmes (e.g. IEC or WASH) that are introduced during the study period.

All parasitological data will be double-entered and cross-checked by experienced data entry clerks employed at CSRS. Statistical analyses will be carried out using EpiInfo version 3.5 (Centers for Disease Control and Prevention; Atlanta, United States of America) and STATA version 10 (Stata Corp.; College Station, United States of America). The primary outcome will be the change in prevalence and intensity of *S. haematobium* infection in each of the four study population groups over the 3 years of the study. Each year, *S. haematobium* prevalence and infection intensity data will be calculated. The results from the different study arms will be compared on an annual basis and at the end of the 3-year intervention period. The snail abundance and infection rates over time will allow us to draw inference on the force of transmission. Moreover, the overall impact of the different treatment schemes on soil-transmitted helminthiasis will be measured in terms of prevalence and intensity of infection.

#### Minimise bias

There are a host of factors that could affect the outcomes, including political instability as experienced in our previous and still ongoing SCORE-funded research [24]. However, our team is well prepared for such issues. Maintaining good communication among the partners and the national schistosomiasis control programme, local authorities and the SCORE secretariat will minimise potential negative outcomes.

### Protocol review and ethical clearance

The study protocol has been approved by the institutional research commissions of CSRS in Abidjan and Swiss TPH in Basel. Ethical approval was obtained from the ethics committees in Switzerland: “Ethikkommission Nordwest- und Zentralschweiz (EKNZ)” (reference no. UBE-15/34; date of approval 15 April 2015) and in Côte d’Ivoire: Comité National d’Éthique et de la Recherche, Ministère de la Santé et de Lutte contre le SIDA (reference no. 007/MSLS/CNER-kp; date of approval 2 February 2016) and Direction Générale des Productions et de la Sécurité Alimentaire, Ministère de l’Agriculture, (reference no. 0163/MINAGRI/DGPSA/DPVCQ; date of approval 27 January 2015). The trial is registered at the International Standard Randomised Controlled Trial Number (ISCRCTN) Register (ISRCTN10926858; http://www.controlled-trials.com/ISRCTN10926858; date of registration 21 December 2016).

Written informed consent will be sought from adults and parents or legal guardians of children below the age of 18 years. Children aged between 5 and 18 years will sign an informed assent form. Participation is voluntary and study participants have the right to withdraw from the study at any given point in time with no further obligations.

The results of this study might be published, but the names of subjects or their identities will not be revealed. Records will remain confidential and will be stored in a secured locker. The results of tests will be coded to prevent association with participants’ names. The investigators will keep a separate confidential enrolment log that matches identifying codes with the subjects’ names. Only authorized personnel directly involved in the study will have access to data entered into computerized files and encoded. Subject-specific information may be provided to medical personnel only with the subject’s permission.

Depending on the intervention arm, subjects will be treated free of charge once or twice a year with praziquantel (40 mg/kg) using a dose-pole [9, 49] and with a single dose of albendazole (400 mg). Adverse events will be monitored and recorded as detailed before. Of note, praziquantel and albendazole are considered safe and well-tolerated drugs, which are annually distributed to millions of people in endemic countries during MDA campaigns in line with WHO guidelines. In Côte d’Ivoire, such treatment campaigns have been commenced by the PNLMTN-CP, which is also in charge of the MDA in the frame of this project. No potential risk is linked to participation in the project as the collected samples are limited to stool and urine.

## Discussion

In some regions of Côte d’Ivoire, elimination of schistosomiasis seems feasible, due to a strong seasonality of the disease transmission. In line with WHO recommendations published in 2012 [30], the country now aims to interrupt the transmission of *S. haematobium* infections in some selected areas in the central and northern regions. Our study will reveal whether or not urogenital schistosomiasis transmission can indeed be interrupted in the targeted areas in northern and central parts of Côte d’Ivoire with a package of well-tailored and focussed interventions implemented over a 3-year period. It will also show whether it is feasible to control soil-transmitted helminthiasis as a public health problem in the targeted areas. Finally, it will elucidate whether annual MDA with praziquantel and albendazole before the peak transmission season of urogenital schistosomiasis (arm A) or annual MDA with praziquantel and albendazole after the peak transmission season of schistosomiasis (arm B) are sufficient measures to interrupt transmission of urogenital schistosomiasis or whether intensified interventions such as two yearly MDAs before and after the peak schistosomiasis transmission (arm C) or annual MDA with praziquantel and albendazole before the peak schistosomiasis transmission season, coupled with chemical snail control using niclosamide (arm D) are needed to break transmission.

As several cases of reinfection with *S. haematobium* following exclusive chemotherapy have been reported in Côte d’Ivoire [50, 51] and elsewhere [52–54], we hypothesise that MDA timed with chemical snail control with niclosamide will be the best measure for elimination of urogenital schistosomiasis transmission. If confirmed in our study, this approach might be adopted by the PNLMTN-CP in its strategic plan for controlling and eliminating schistosomiasis from Côte d’Ivoire in the next several years. Of note, MDA plus snail control has been shown to contribute to the interruption of schistosomiasis transmission in other countries [20, 55, 56]. This approach is currently being applied, and its impact assessed, in a multi-year project to eliminate urogenital schistosomiasis transmission on the Zanzibar archipelago [37, 46]. While there is no evidence about tolerance or resistance to niclosamide in snails thus far [57, 58], intensive application of niclosamide will require careful monitoring of snails and other non-target fauna.

The study on interrupting seasonal transmission of urogenital schistosomiasis and control of soil-transmitted helminthiasis described here will provide an evidence-base for future programme decisions for an informed choice between exclusive schemes of MDA (according to the type and frequency) and MDA combined with chemical snail control for interrupting seasonal transmission of urogenital schistosomiasis in areas with marked seasonality as found in northern and central parts of Côte d’Ivoire. With this new insight into disease elimination approaches, the study will help to improve guidelines for neglected tropical disease control and elimination programmes and decision makers in Côte d’Ivoire and in other endemic countries.

## Abbreviations

CDD: Community drug distributor
CSRS: Centre Suisse de Recherches Scientifiques en Côte d’Ivoire
CWT: Community-wide treatment
DALY: Disability-adjusted life year
EKNZ: Ethikkommission Nordwest- und Zentralschweiz
GNC: General national census
GPS: Global positioning system
IEC: Information, education and communication
ISCRCTN: International Standard Randomised Controlled Trial Number
MDA: Mass drug administration
MSc: Mollusciding
NHM: Natural History Museum
PNLMTN-CP: Programme National de Lutte contre les Maladies Tropicales Négligées à Chimiothérapie Préventive
POC-CCA: Point-of-care circulating cathodic antigen
SBT: School-based treatment
SCAN: Schistosomiasis Collection at the NHM in London
SCI: Schistosomiasis Control Initiative
SCORE: Schistosomiasis Consortium for Operational Research and Evaluation
Swiss TPH: Swiss Tropical and Public Health Institute
UFHB: Université Félix Houphouët-Boigny
WASH: Water, sanitation and hygiene
WHO: World Health Organization
ZEST: Zanzibar Elimination of Schistosomiasis Transmission
